# Author Correction: Dynamic regulation of canonical TGFβ signalling by endothelial transcription factor ERG protects from liver fibrogenesis

**DOI:** 10.1038/s41467-020-15151-w

**Published:** 2020-03-05

**Authors:** Neil P. Dufton, Claire R. Peghaire, Lourdes Osuna-Almagro, Claudio Raimondi, Viktoria Kalna, Abhishek Chauhan, Gwilym Webb, Youwen Yang, Graeme M. Birdsey, Patricia Lalor, Justin C. Mason, David H. Adams, Anna M. Randi

**Affiliations:** 1 Vascular Sciences, Imperial Centre for Translational and Experimental Medicine, National Heart and Lung Institute, Imperial College London, London, W12 0NN UK; 2 Centre for Liver Research, Institute of Biomedical Research, Institute of Immunology and Immunotherapy, College of Medical and Dental Sciences, University of Birmingham, Birmingham, B15 2TT UK

**Keywords:** Cell signalling, Transcriptional regulatory elements, Mechanisms of disease, Liver fibrosis

Correction to: *Nature Communications* 10.1038/s41467-017-01169-0, published online 12 October 2017.

The original version of this Article contained an error in the spelling of the author Abhishek Chauhan, which was incorrectly given as Abhishek Chuahan. This has now been corrected in both the PDF and HTML versions of the Article.

