# Supplementary material for: Dynamic regulation of canonical TGFβ signalling by endothelial transcription factor ERG protects from liver fibrogenesis
Source: Nat Commun. 2017 Oct 12;8:895. doi: 10.1038/s41467-017-01169-0 (PMC5638819; doi:10.1038/s41467-017-01169-0)
Supplement: Supplementary file 1 — Supplementary Information [file 41467_2017_1169_MOESM1_ESM.pdf]

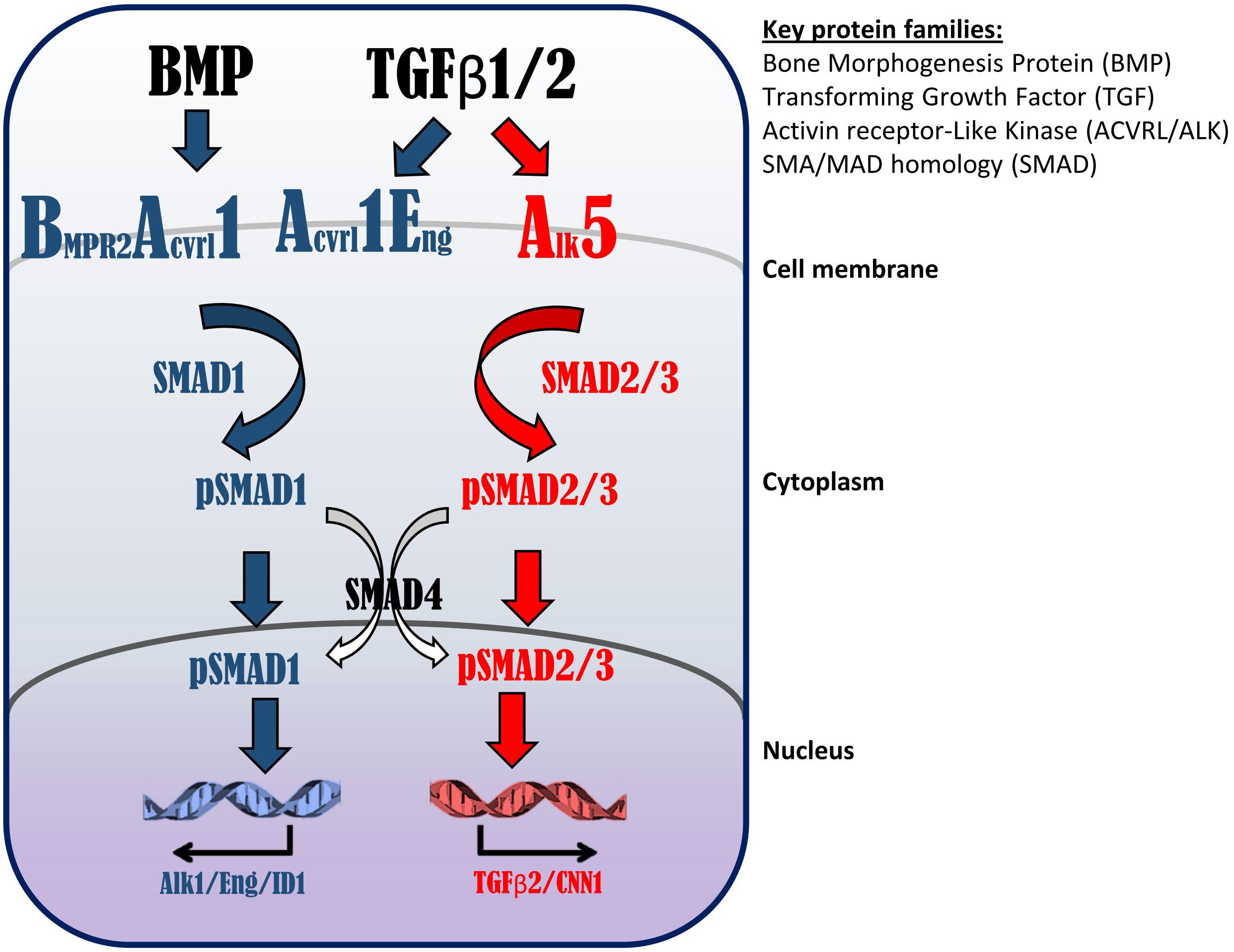

Supplementary Figure 1. ***Schematic of the SMAD-dependent TGF/BMP canonical signalling pathways in EC***
Schematic of the SMAD-dependent TGF/BMP canonical signalling pathways in EC with abbreviations of protein families involved in canonical signalling.

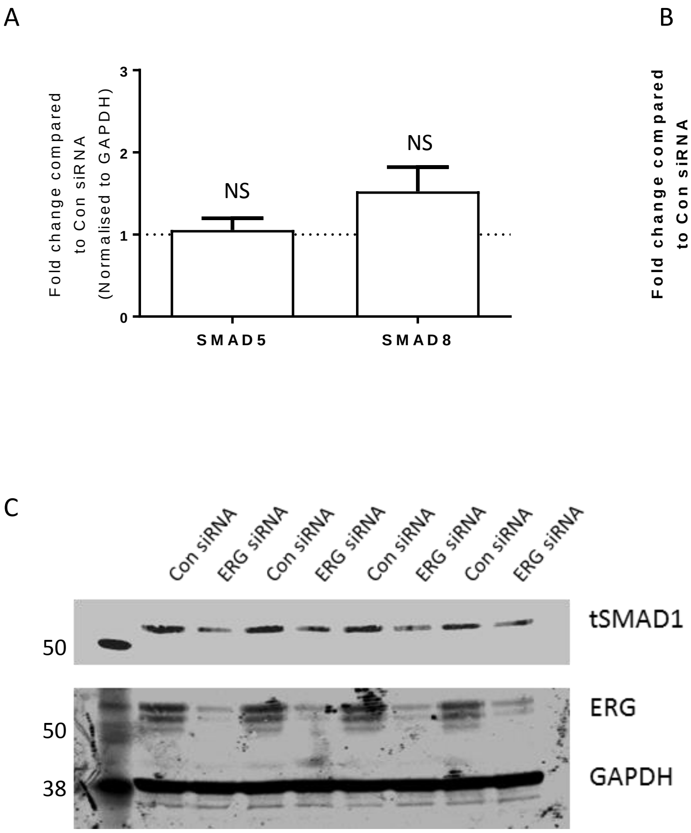

Supplementary Figure 2. ***Validation of ERG regulation of gene and protein expression of genes involved in the TGF/BMP canonical signalling pathway.***

*(A) ERG does not regulate transcription of additional signalling SMAD proteins (n=3). (B) A second ERG siRNA (#2) was used to confirm profile of key target genes (n=3). (C) SMAD1 protein expression was assessed following 48 h transfection in individual HUVEC donors with Control (Con) or ERG siRNA (n=4). Quantified by fluorescence intensity was normalized to GAPDH for SMAD1 and provided in Fig. 1D. Data normalised to GAPDH and compared to control siRNA (*) treated by unpaired T-test. All graphical data are mean ± s.e.m, *P < 0.05, **P <0.01, ***P < 0.001.*

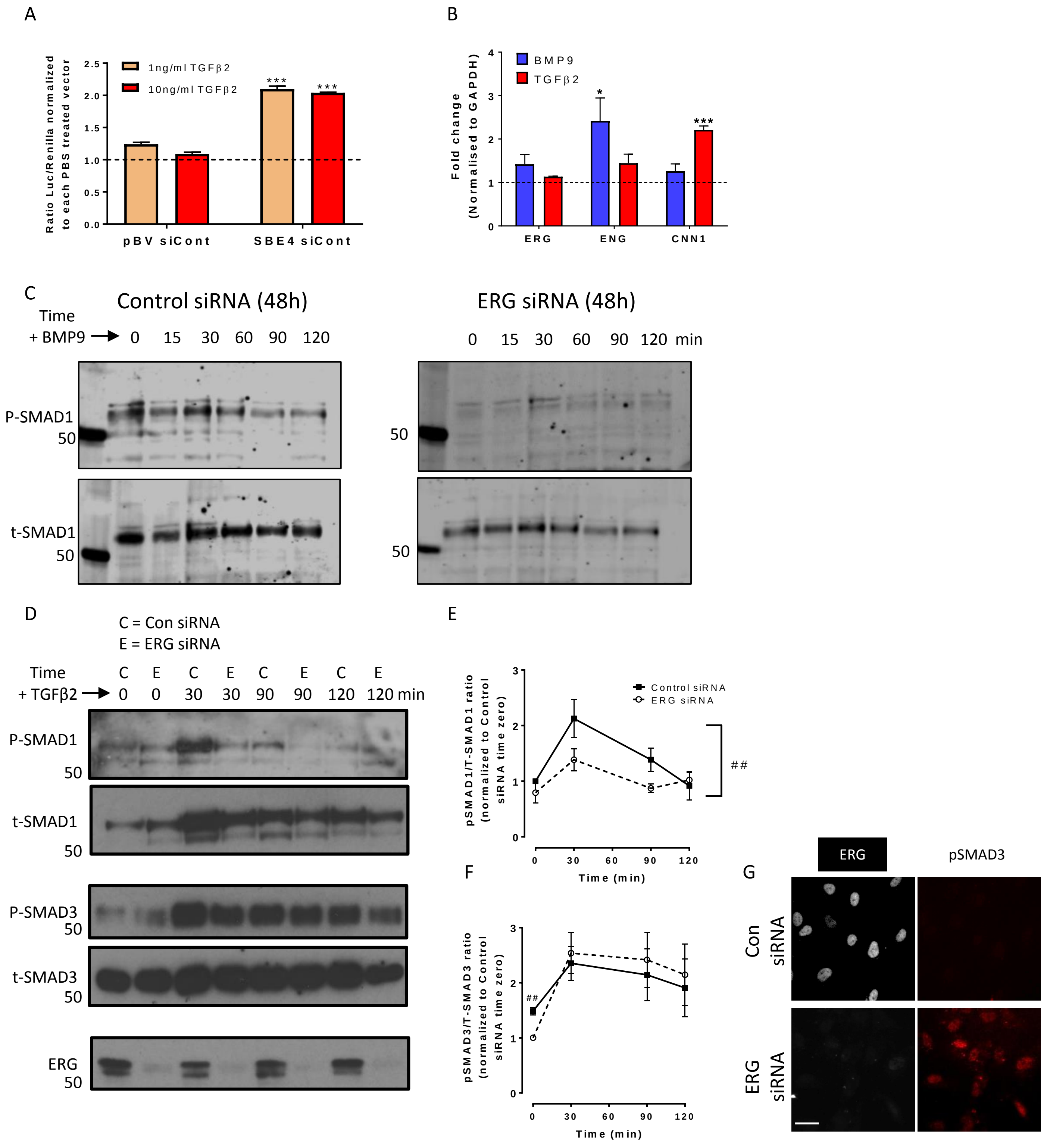

Supplementary Figure 3. ***Representative western blots of SMAD signalling in control siRNA and ERG siRNA treated HUVEC***

*(A) SMAD3 transactivation with two TGFβ2 concentrations was assessed using SMAD3 reporter pBV-SBE4 luciferase reporter assay. The ratio of luciferase to renilla signal from each transfection was determined and normalized to empty vector (EV). (B) Ligand specificity following BMP9 (1 ng ml^{-1}) or TGFβ2 (10 ng ml^{-1}) treatment was assessed by qPCR for specific SMAD1-associated (ENG) or SMAD3-associated (CNN1) target genes. Representative immuno-blots following (C) BMP9 (1 ng ml^{-1}) or (D) TGFβ2 (10 ng ml^{-1}) treatment were performed for SMAD1, SMAD3 and ERG over a 2 h time course in control and ERG siRNA HUVEC (transfected for 48 h). Phosphorylation of (E) SMAD1 or (F) SMAD3 was normalised to total protein following TGFβ2 (10ng ml^{-1}) (n=4). Control and ERG siRNA treated HUVEC were analysed at individual time points. (G) Immunofluorescence was used to confirm basal pSMAD3 phosphorylation in ERG siRNA treated HUVEC. Scale bar 20 µm. Data was compared to untreated (*) or to control siRNA treated (#) by unpaired T-test. Curves were analysed by two-way ANOVA compared to control siRNA (#). All graphical data are mean ± s.e.m, * or # P < 0.05, ** or ## P <0.01, *** or ### P < 0.001.*

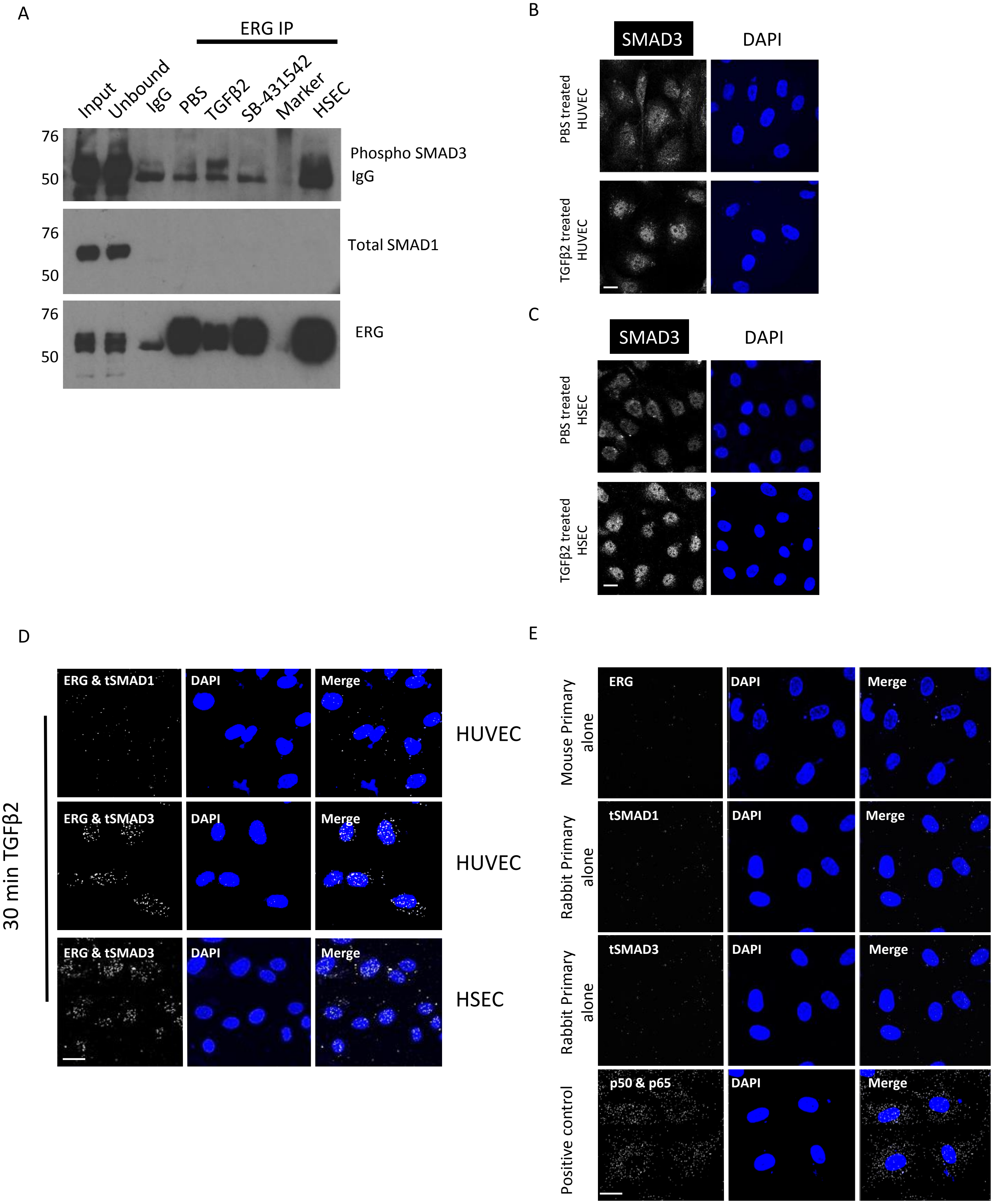

Supplementary Figure 4. ***Co-immunoprecipitation and Proximity ligation assay for ERG and SMADs.***
(A) Representative Co-IP experiment performed by ERG pull-down in HUVEC treated with PBS, TGFβ2 (10ng ml^{-1}; 30 min) or pre-treated with SB-542431 (10 µM; -1h) and in HSEC under control conditions (n=3). Translocation of SMAD3 to the nucleus was assessed following TGFβ2 stimulation (10ng ml^{-1}) for 30 min in (B) HUVEC and (C) HSEC fixed with 4% PFA and stained with rabbit α-SMAD3 (white) and DAPI (blue). All scale bars 20 µm. (D) PLA assay performed following stimulation with TGFβ2 (10ng ml^{-1}; 30 min) for ERG and tSMAD1 (top panel; HUVEC) and ERG and tSMAD3 in HUVEC (middle panel) and HSEC (bottom panel). (E) Background PLA assay signal was established by assessing each primary antibody alone; a positive control signal was assessed using NFκB sub-units p50 with p65 in unstimulated HUVEC.

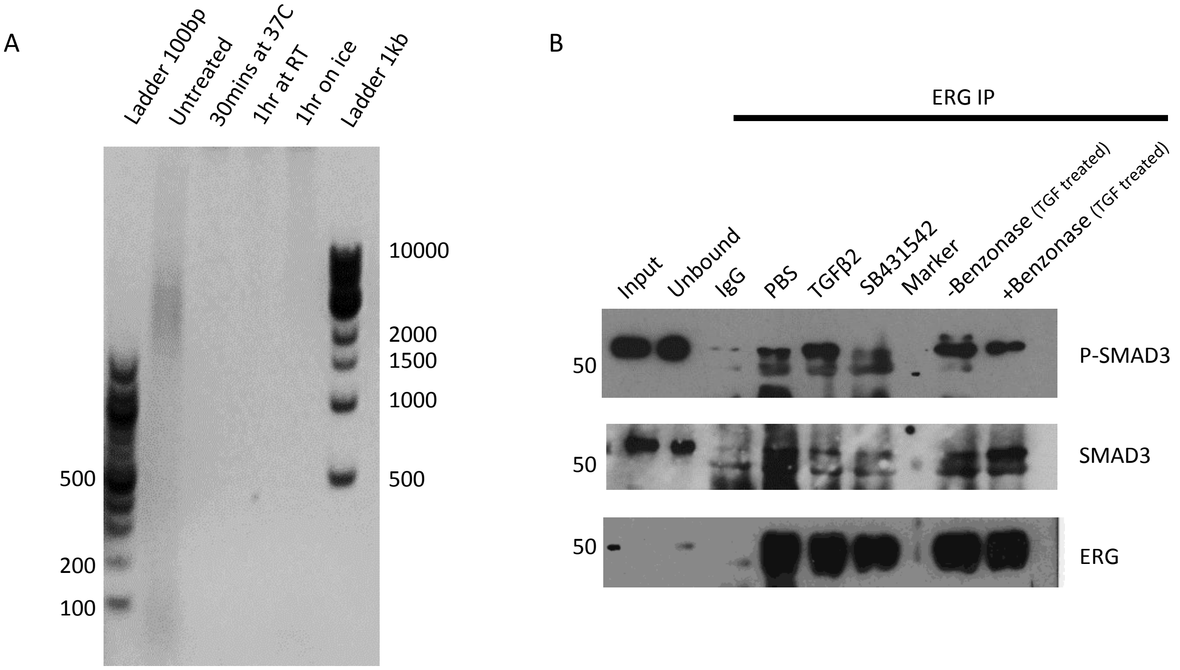

Supplementary Figure 5. ***Interaction between ERG and SMAD3 does not require DNA.***

To assess the requirement for DNA to preserve the complex between ERG and SMAD3, whole lysates were treated with Benzonase (500 units) prior to Co-IP experiment performed by ERG pull-down. (A) Efficacy of Benzonase to remove DNA from protein lysates was tested to obtain optimal conditions (1 h RT) for (B) co-immunoprecipitation (representative of n=2).

A

TGFβ2 ChIP-PCR region with ERG and SMAD motifs

```
>hg19_dna range=chr1:218518168-218518967 5'pad=0 3'pad=0
repeatMasking=none
ACAACAAACTAACAAAATAAATTCTGGGATGGCAACCTGCTAAGGTATCC -461
CAGAAAATAAGAGGTAGGACATGAATTTAAAAGATTGGAAGGTATGTCTT
CAGTACTGGCCTGGCCCTGAGTAGACTAGTGCTCCCTCCCATAGGGGTGC -361
GTGTGCACACATAATACAGGAGGGAAGCCTTCCCTTCTAGAGCAAGTGAT
TCAGCTTGGGAGGCTGTGACTGAGCTACACTAAGTAAAAACGGGAGACTT -261
GATTGTCCTTCCTTCAACAGACCTGTCCAAAATGACTGGAAAGTAAATAC
CGTAAATCACTGTTGTCAGGGCGCACATTCCACCTCCTTCCTCCCTTACC -161
CACAGCGGTCCTCATTTCCACACTCCCTCAACGGTTCGGGGAGAGCTCGT
GGTCTAAGTAACGAGAGGACTTCTGACTGTAATCCTAGCACGTCACTTTG -61
TTGAAGGCAGACACGTGGTTCAGAGAGAACTTATAAATCTCCCCTCCCCG
GCAAGATCGTGATGTTATCTGCTGGCAGCAGAAGGTTCGCTCCGAGCGGA +38
GCTCCAGAAGCTCCTGACAAGAGAAAGACAGATTGAGATAGAGATAGAAA
GAGAAAGAGAGAAAGAGACAGCAGAGCGAGAGCGCAAGTGAAAGAGGCAG +138
GGGAGGGGGATGGAGAATATTAGCCTGACGGTCTAGGGAGTCATCCAGGA
ACAAACTGAGGGGCTGCCCGGCTGCAGACAGGAGGAGACAGAGAGGATCT +238
ATTTTAGGGTGGCAAGTGCCTACCTACCCTAAGCGAGCAATTCCACGTTG
```

Key: ERG binding sites: Blue
SMAD binding element (SBE): Green and Yellow
Primer targeting region: Grey

B

CNN1 ChIP-PCR region with ERG and SMAD motifs

```
>hg19_dna range=chr19:11646624-11647573 5'pad=0 3'pad=0
repeatMasking=none
CTACCCCCATCTACCAGACAGAGACGCGAAGAGAGACGGACAATACAGCG -2977
ACAGCGACATGAAGAGAAACAGAATGACTCAAAGACCAGGCACAGAAACA
GAGACACAGAGACAAAGGCAGAGACAGAAAAAGGCTCAGCGACCCAGAGA -2877
CGGAGATACAGAGACACAGACCTACTACAATCAGAGGGACAAGGCAGTCC
CCGAAAGGGCTGCCCCCTGGGGGTACCCGGGGCTGCCCCGTTGGGGGGCA -2777
CGCCAAGAGCTCCCCCACCCCGCCCCCGTCGTGCCTGGGCCGTGACTTCA
GAGTCCGTGCAGATTCTATAAATGGCCAAGAGGCCGCGGGCCGGGGGGCG -2677
GGGCCGAGTGAGTCATCGCGTGACGTCACCGCCGCCTGGACCTCGCCAGG
TCCCGCCCCCGCCCCTCCCGGGCCGCCAGGGGGCGCCGGCTGCAGGTCCT -2577
CACATGCGCAGTAGCGAACTGCGGCCCCCGAGGGCCGCTCAGTTTGGGAG
GGATCCAGGAATCTGCGCGTTCGAATCCCGCCCCTGCCACCCTCGAGCTG -2477
TGTGACCCGGCCATTCGGCGTCACCTCTCTGTGCCTCGTATAGACCCACC
TCATAAGGCTGTCGTGAGGAATGAGTTAACAGGCTCAAAGCTCTTAGTAC -2377
GTAGCAGGTGCTAATTGTTAGCTCATTTTATGTACTTTCTATTTTTTCTT
TTTTTTTTTTTTAAATAGAGACGGGGTCTCGCTATGTTGCCTAAGCTGGT -2277
CTCGGGTCTCGAACTCCAGGGCTCAAGCGATCCTCCCGCCTTGGCCTCGT
ATTTTATGTATTTTCGTGTATGTTCAACATATCATGACATGCCATCAACT -2177
AGCACATTGCCTTAGCTCAGTGGTTGGCAAACAGGGGTGATCTGCTTCCA
CCCCGACCCCAATCATGGGACATTGGCAATGTGTGTAGACATTTCTTCTT -2077
```

C

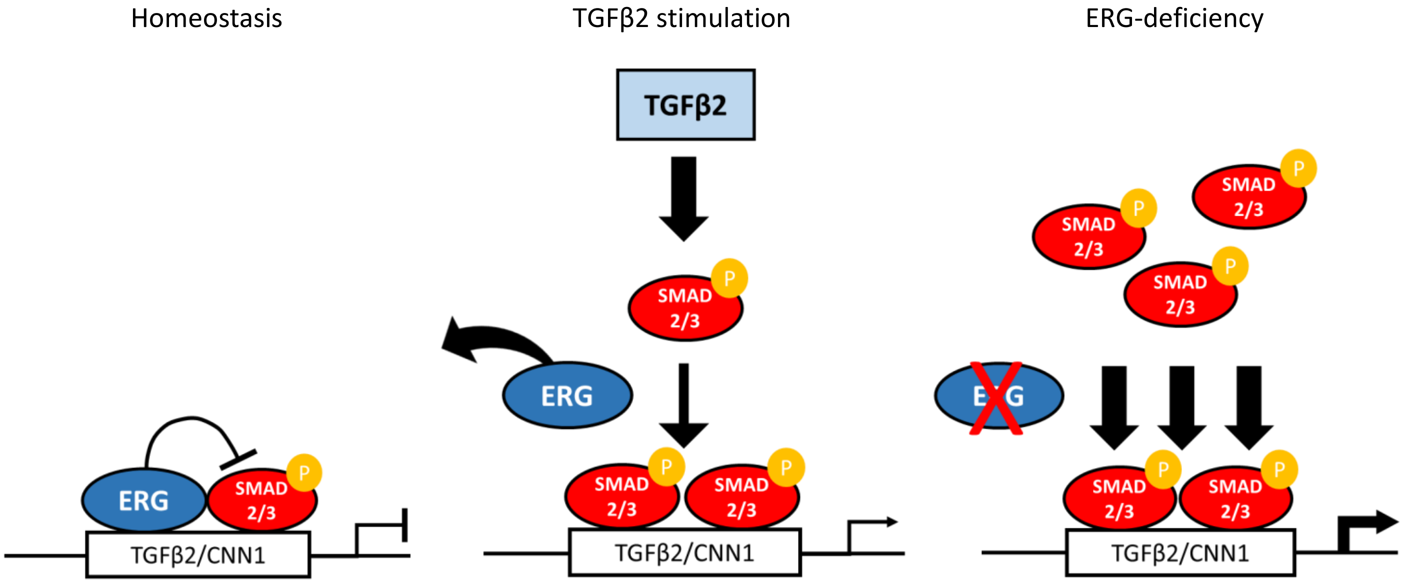

Supplementary Figure 6. ***Putative ERG and SMAD motifs within TGFβ2 and CNN1 promoter regions***
DNA sequences corresponding to the regions targeted for ChIP-PCR within (A) TGFβ2 and (B) CNN1 genes were obtained from UCSC browser and annotated for ERG motifs (blue) and the two known SMAD binding motifs (yellow and green); primer-spanning regions in grey. (C) Schematic model of ERG regulation of SMAD2/3 signalling: in basal conditions (left panel) ERG binds and represses SMAD3-driven EndMT gene expression. Upon TGFβ2 stimulation (middle panel), ERG is displaced from DNA by SMAD3. Loss of ERG (right panel) also results in enhanced SMAD3 binding to its target DNA regions on TGFβ2 and CNN1 promoters.

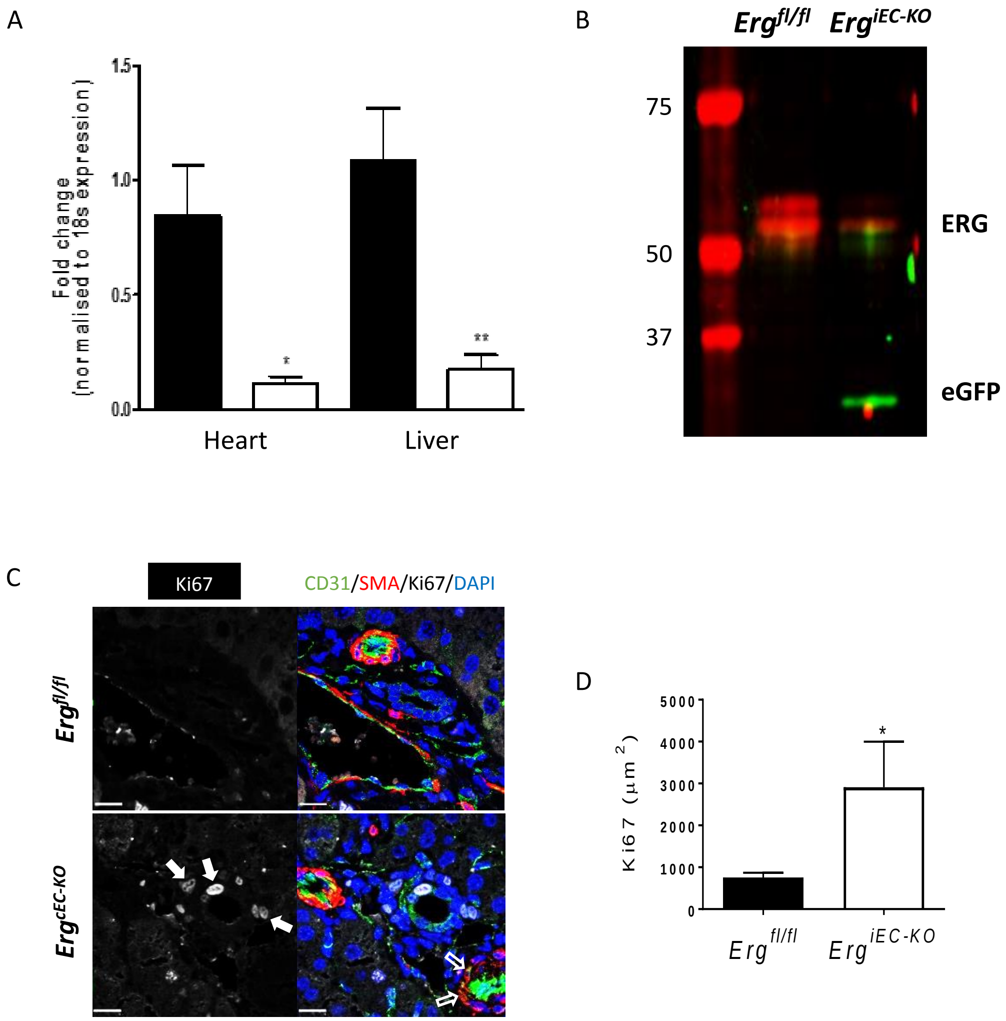

Supplementary Figure 7. ***Characterisation and phenotype of Erg^{iEC-KO} mice***
*(A) qPCR for ERG was carried out on tamoxifen-treated Erg^{iEC-KO} and Erg$^{fl/fl}$ mice to assess ERG expression in heart and liver tissue. (B) Representative western blot analysis of ERG and eGFP expression from heart protein lysates. (C) Representative image of Ki67 expression (white; filled arrows) in biliary cells in portal tracts, with CD31 (green) and SMA (red). CD31$^+$SMA$^+$ double positive cells (open arrows merged panel) (D) quantification of Ki67$^+$ area (3 fields per mouse, n=3; scale bar 20μm). Data was compared to littermate Erg$^{fl/fl}$ littermate controls (*) by unpaired T-test. All graphical data are mean ± SEM, *P < 0.05.*

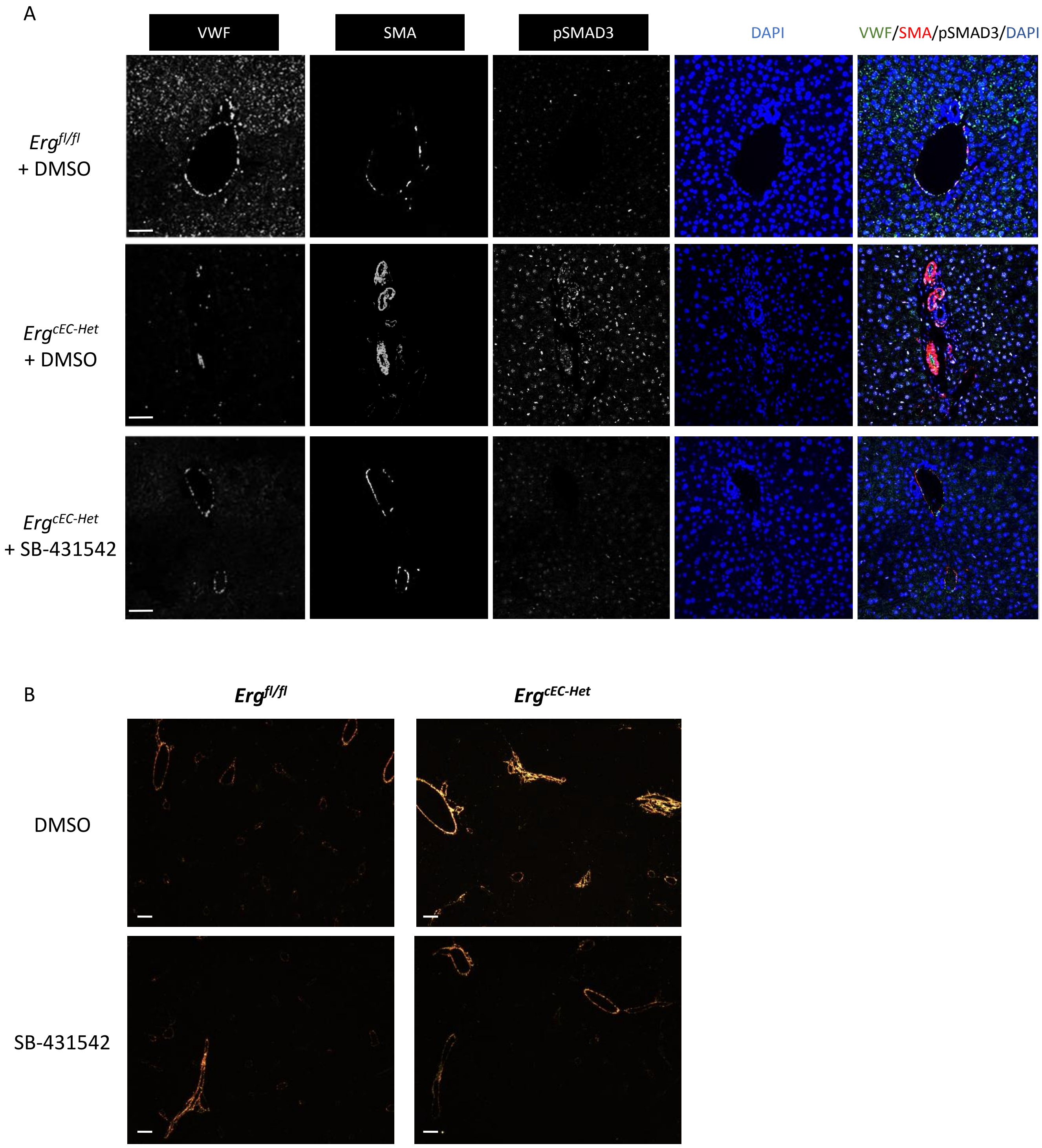

Supplementary Figure 8 ***Inhibition of pSMAD3 and collagen deposition by in vivo administration of SB-431542***
(A) Representative immunofluorescence images of pSMAD3 staining in portal tract region of DMSO-treated Erg$^{cEC\text{-}het}$ and SB-431542 (10mg kg^{-1})-treated Erg$^{cEC\text{-}het}$. Injections were performed three times a week for 2 weeks. (B) Representative images of picro-sirius red staining quantified in Figure. 5E. All scale bars 50 µm.

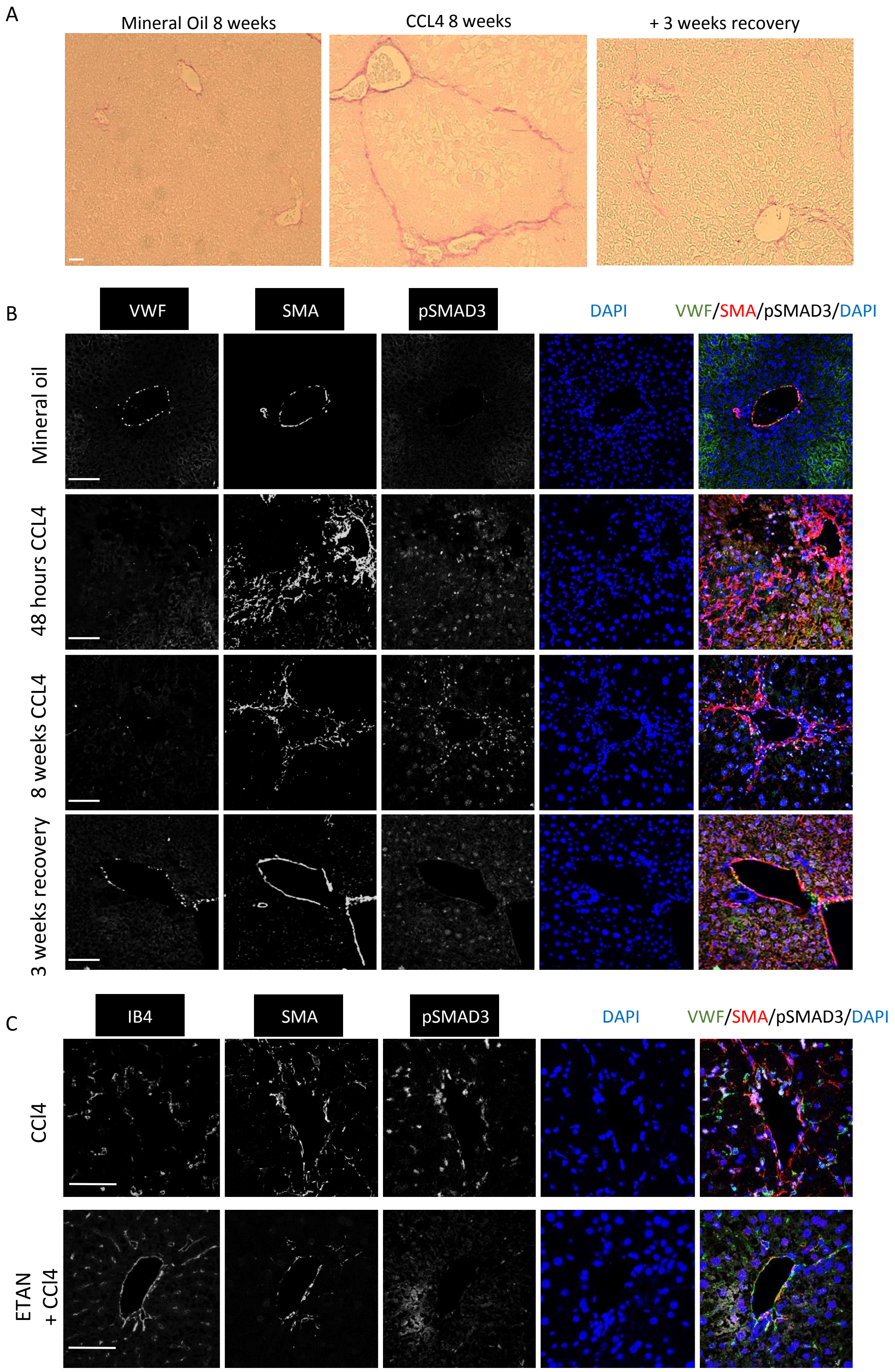

Supplementary Figure 9 ***Immunohistochemically analysis of CCL_4 treatment performed in WT mice***

(A) Representative bright field images of picro-sirius red staining following mineral oil, chronic CCL_4 administration or following a 3-week recovery period following chronic CCL_4 administration. (B) pSMAD3 staining in portal tract region by immunofluorescence for acute or chronic administration of CCL_4 or following the subsequent recovery period. (C) Representative images of pSMAD3 staining following co-administration of etanercept and CCL_4 for 48h quantified in Figure. 6H. All Scale bars 50 µm.

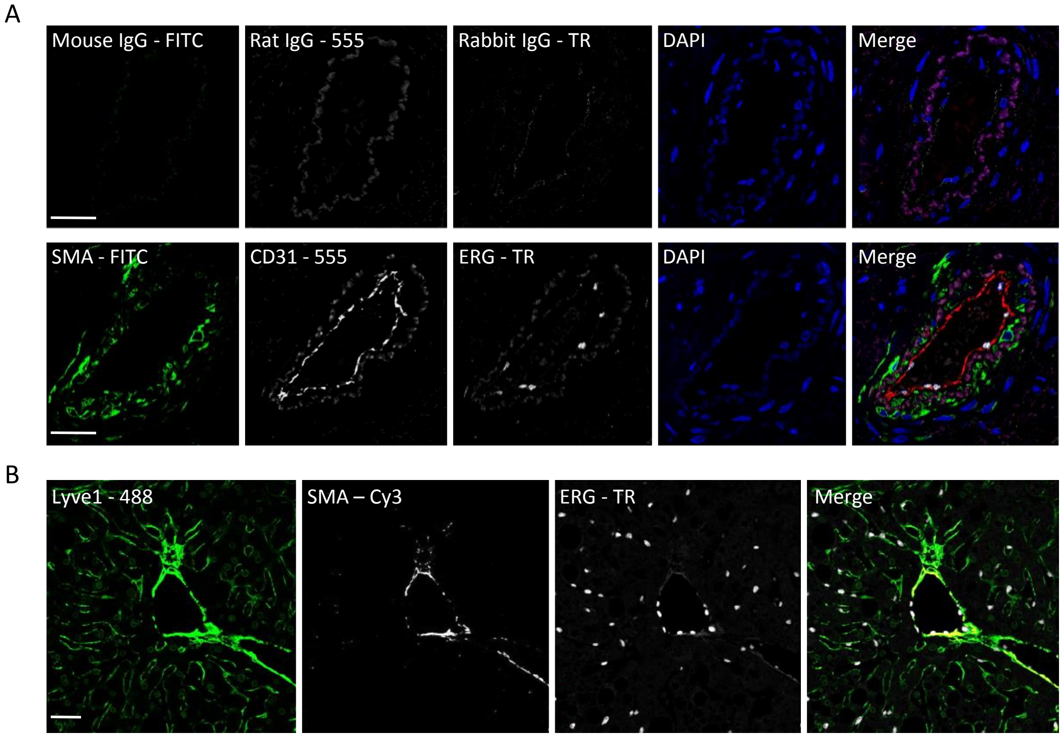

Supplementary Figure 10 ***Characterisation of ERG expression in non-fibrotic human liver sections***
(A) Isotype control staining for SMA (green), CD31 (greyscale; red in merge) and ERG (white) conducted in parallel with corresponding IgG antibodies. (B) Confirmation of ERG expression in sinusoidal EC was assessed by mouse α-Lyve1 (green) and SMA (greyscale; red in merge). All scale bars 20 µm.

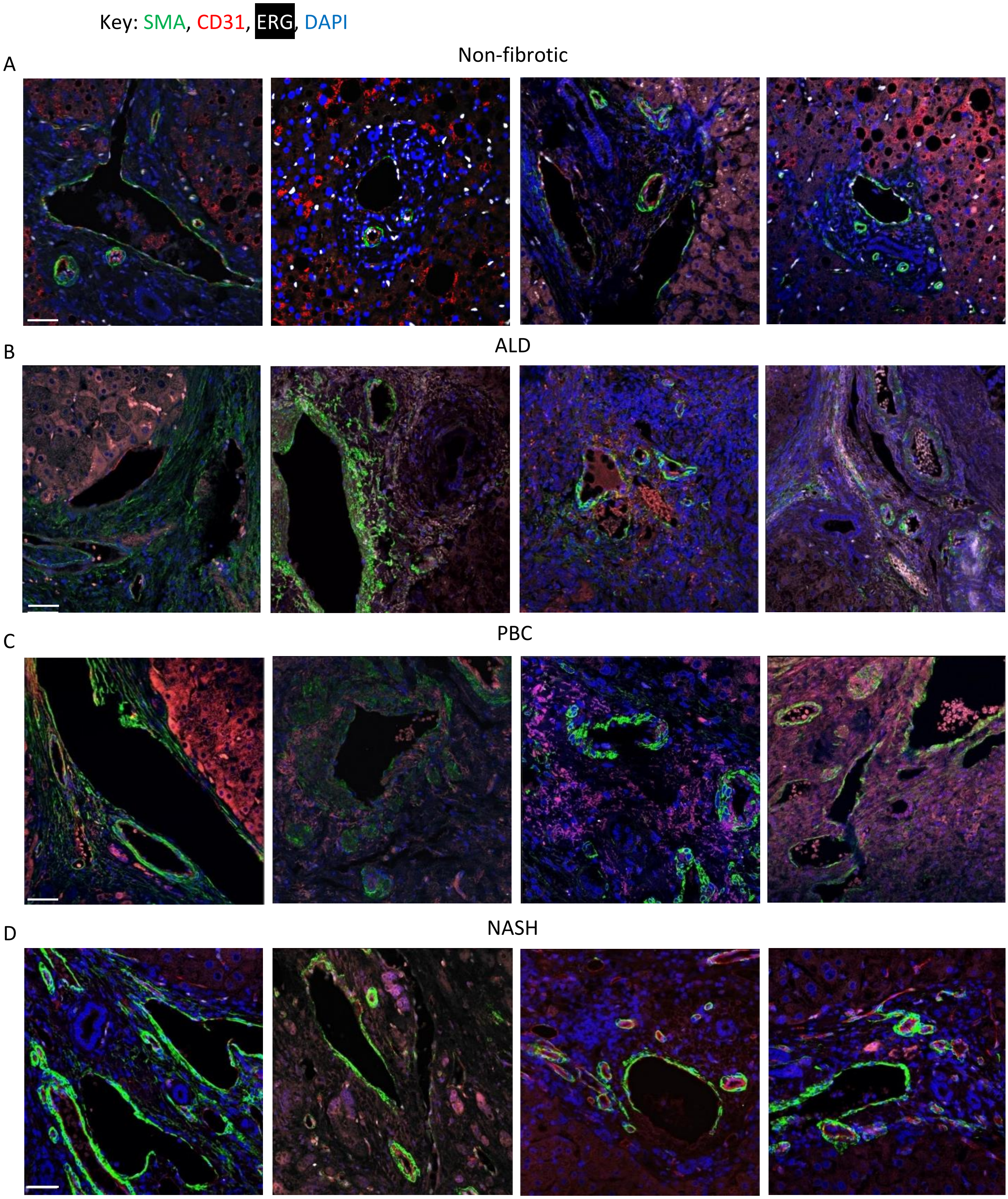

Supplementary Figure 11. ***Representative portal tracts from human tissue sections***

Each panel is a representative merged image from an individual patient liver sample stained for SMA (green), CD31 (red), ERG (white) and DAPI (blue) within each group. (A) Non-fibrotic (B) ALD (C) PBC and (D) NASH. All scale bars 50 µm.

Figure 1C

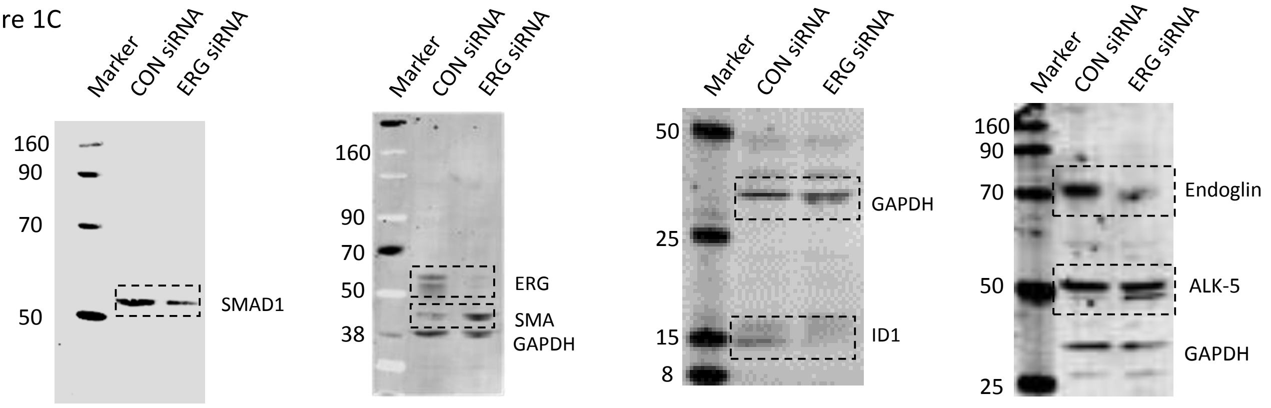

Supplementary Figure 3D

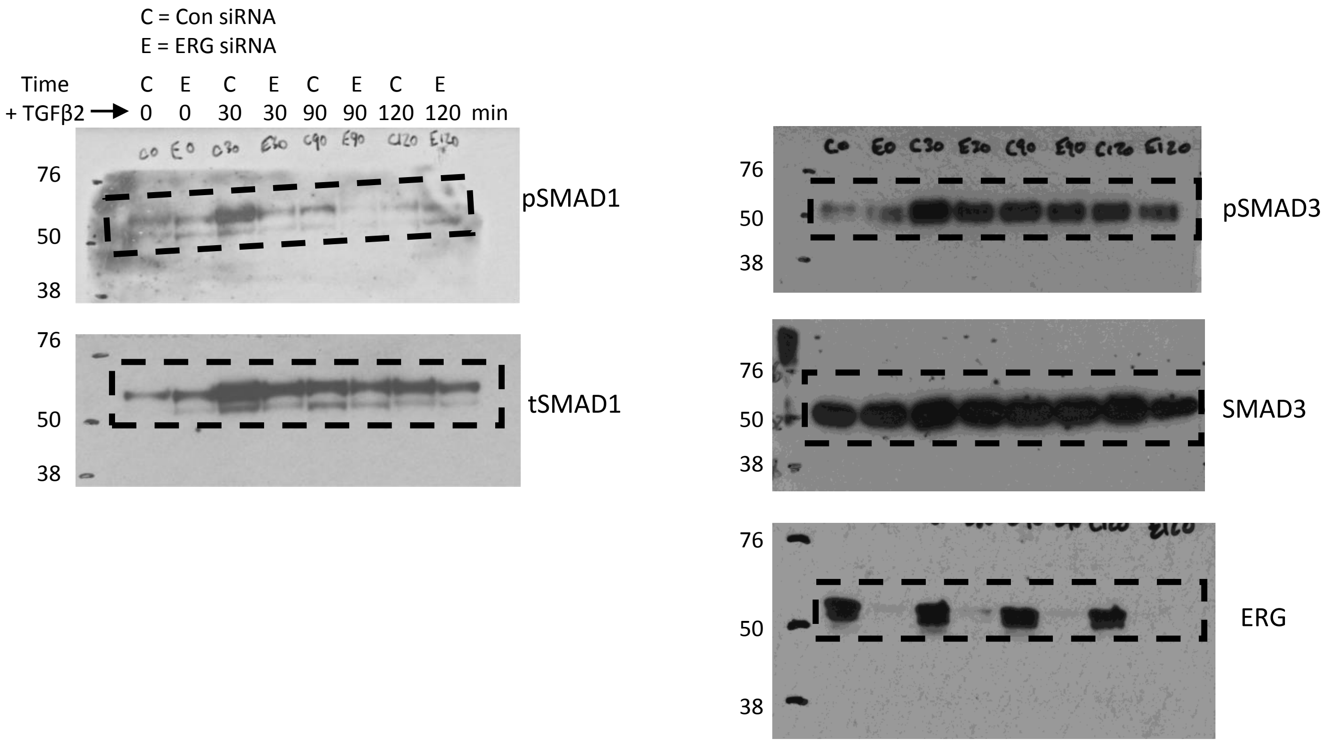

Supplementary Figure 12 **Uncropped Western blotting images for figures**

Figure. 2F

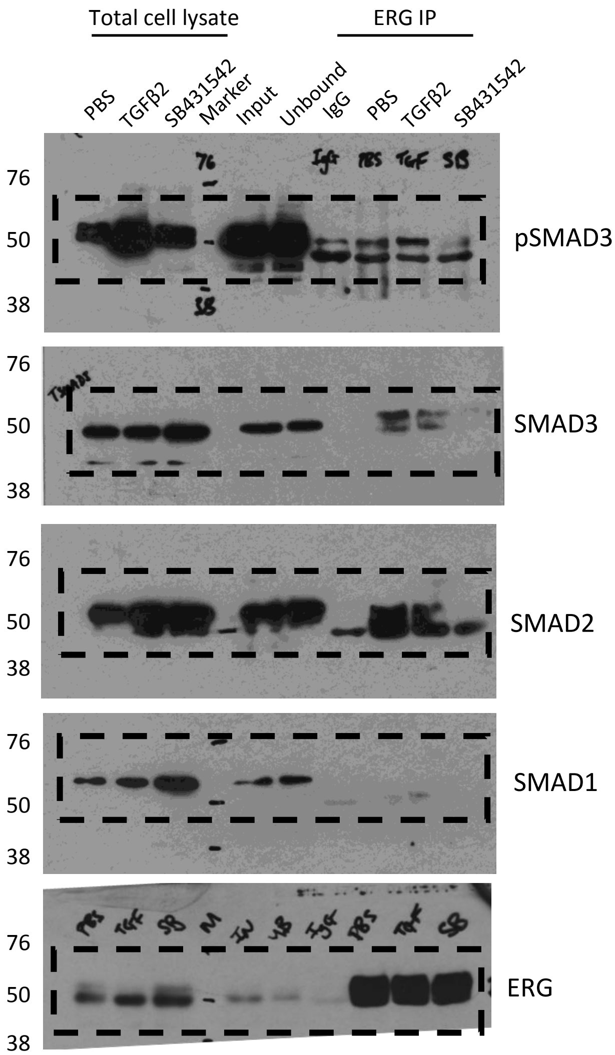

Supplementary Figure 4A

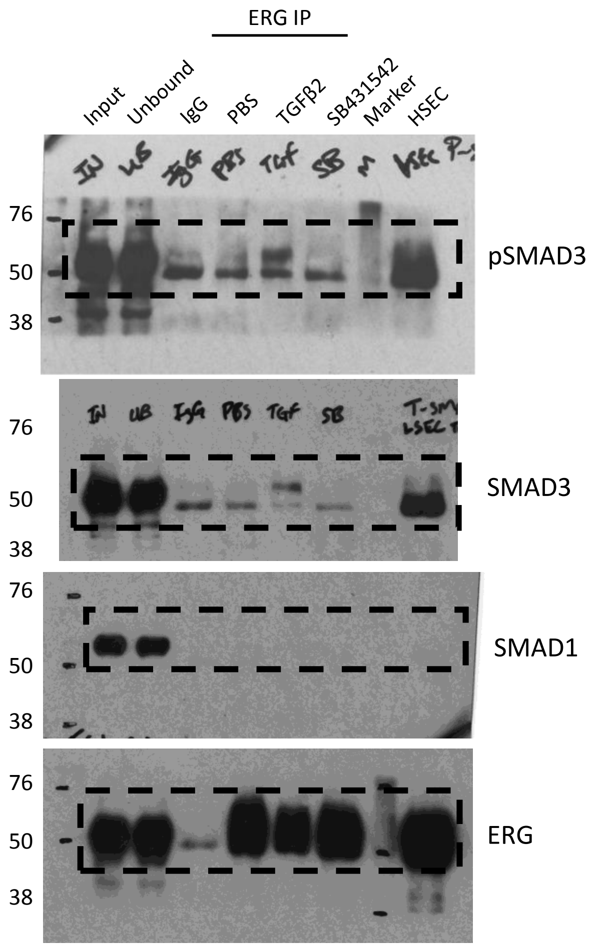

Figure 2G

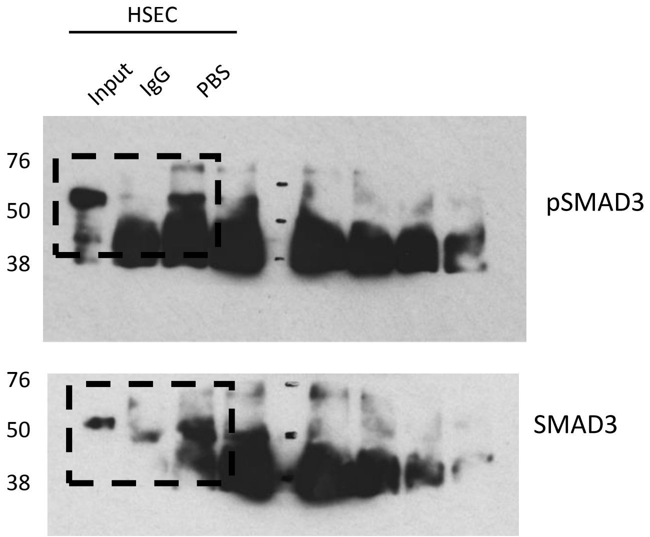

Supplementary Figure 5B

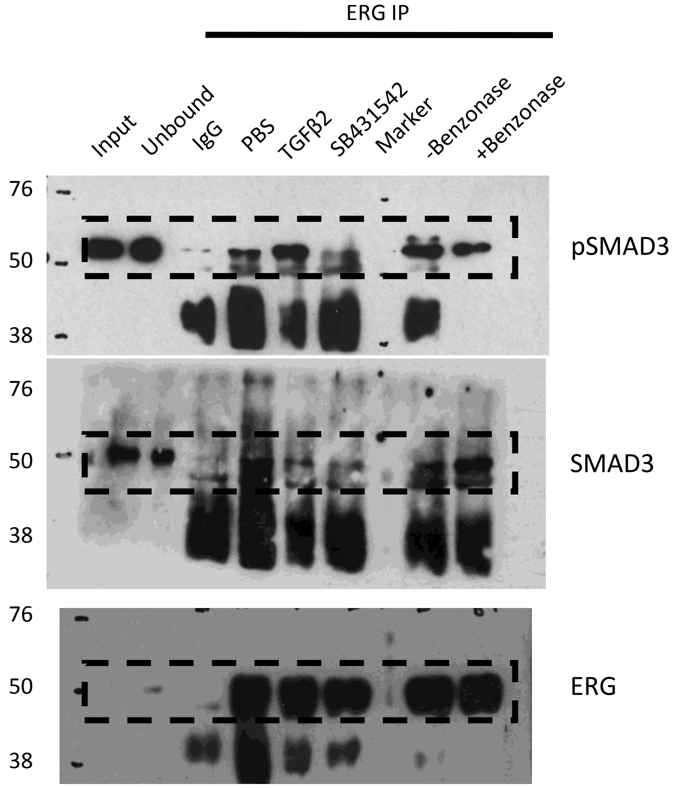

Supplementary Figure 13 **Uncropped Co-IP Western blotting images for figures**

OFFICIAL GENE SYMBOL	GENE NAME	Driven or Repressed
ACVRL1 (ALK1)	activin A receptor like type 1(ACVRL1)	Driven #
AKT3	AKT serine/threonine kinase 3(AKT3)	Driven
ATR	ATR serine/threonine kinase(ATR)	Repressed
BMP2	bone morphogenetic protein 2(BMP2)	Repressed
BMP6	bone morphogenetic protein 6(BMP6)	Driven
BMPR2	bone morphogenetic protein receptor type 2(BMPR2)	Driven #
CD79B	CD79b molecule(CD79B)	Repressed
CDKN1A	cyclin dependent kinase inhibitor 1A(CDKN1A)	Repressed
CDKN1B	cyclin dependent kinase inhibitor 1B(CDKN1B)	Repressed
COL1A1	collagen type I alpha 1 chain(COL1A1)	Repressed
DAB2	DAB2, clathrin adaptor protein(DAB2)	Driven
DCN	decorin(DCN)	Repressed
ENG	endoglin(ENG)	Driven #
HMGA2	high mobility group AT-hook 2(HMGA2)	Driven
INHBA	inhibin beta A subunit(INHBA)	Driven
LOXL2	lysyl oxidase like 2(LOXL2)	Driven
LTBP1	latent transforming growth factor beta binding protein 1(LTBP1)	Driven #
MSH3	mutS homolog 3(MSH3)	Driven
MYLK	myosin light chain kinase(MYLK)	Driven
NPPB	natriuretic peptide B(NPPB)	Repressed
NRG1	neuregulin 1(NRG1)	Driven
PARVA	parvin alpha(PARVA)	Driven
PPP2CA	protein phosphatase 2 catalytic subunit alpha(PPP2CA)	Repressed
RAC2	ras-related C3 botulinum toxin substrate 2	Driven
RAF1	Raf-1 proto-oncogene, serine/threonine kinase(RAF1)	Repressed
RARB	retinoic acid receptor beta(RARB)	Repressed
RTN4	reticulon 4(RTN4)	Repressed
SMAD4	SMAD family member 4(SMAD4)	Repressed
SMAD7	SMAD family member 7(SMAD7)	Repressed
SMURF1	SMAD specific E3 ubiquitin protein ligase 1(SMURF1)	Repressed #
SMURF2	SMAD specific E3 ubiquitin protein ligase 2(SMURF2)	Driven
TCF7L1	transcription factor 7 like 1(TCF7L1)	Driven
TGFB1	transforming growth factor beta 1(TGFB1)	Driven #
TGFB2	transforming growth factor beta 2(TGFB2)	Repressed #
TGFBR1	transforming growth factor beta receptor 1(TGFBR1)	Repressed
TGFBR2	transforming growth factor beta receptor 2(TGFBR2)	Driven #
THBS1	thrombospondin 1(THBS1)	Driven
TOB1	transducer of ERBB2, 1(TOB1)	Repressed
USP15	ubiquitin specific peptidase 15(USP15)	Repressed
ZFPM2	zinc finger protein, FOG family member 2(ZFPM2)	Repressed
ZNF521	zinc finger protein 521(ZNF521)	Driven

Supplementary Table 1. Gene ontology analysis of HUVEC 24 and 48 hours post ERG siRNA.

Genes related to TGFβ signalling with a significant pathway enrichment score of 1.3 (DAVID). This list is provided in alphabetical order and annotated to identified genes as either putative ERG-driven or –repressed targets[24]. # denotes targets presented in Fig. 1A and B.

**Antibody (host)**	Company	Cat No.	Application
**ERG (mouse)**	Santa Cruz	sc-376293	IP (3 µg), WB (1:500), PLA (1:200)
**ERG (rabbit)**	Santa Cruz	sc-354x	ChIP (2 µg)
**ERG [EPR3864(2)] (rabbit)**	Abcam	ab133264	IF (human and mouse) (1:200)
**Total Smad3 (rabbit)**	Cell Signalling Technologies	9523S	ChIP (2 µg) WB, PLA (1:200)
**Phospho-Smad3 Ser423/425 (rabbit)**	Cell Signalling Technologies	9520S	WB (1:200)
**Phospho-SMAD3 (rabbit)**	Abcam	ab52903	IF (human and mouse) (1:200)
**Total Smad1 (rabbit)**	Cell Signalling Technologies	9743S	WB, PLA (1:200)
**Phospho-Smad1 Ser206 (rabbit)**	Cell Signalling Technologies	5753S	WB (1:200)
**Id1 (rabbit)**	Santa Cruz	sc-488	WB (1:200)
**Endoglin (rabbit)**	Santa Cruz	sc-20632	WB (1:200)
**TGFβR1 (rabbit)**	Santa Cruz	sc-398	WB (1:200)
**SMA (mouse)**	Sigma Aldrich	C6198	WB, IF (human and mouse) (1:500)
**SMAD1 (mouse)**	Santa Cruz	sc-7965	IF (human and mouse) (1:100)
**VWF-HRP (rabbit)**	Dako	P0226	IF (human and mouse) (1:200)
**CD31 (rat)**	RnD systems	557355	IF (human) (1:100)
**Ki67 (rat)**	eBioscience	14-5698-82	IF (mouse) (1:100)

Supplementary Table 2. Antibodies used in this study

Primers		Oligonucleotide Sequences
**ERG**	Forward	5’-GGAGTGGGCGGTGAAAGA-3’
	Reverse	5’-AAGGATGTCGGCGTTGTAGC-3’
**GAPDH**	Forward	5’-CAAGGTCATCCATGACAACTTTG-3’
	Reverse	5’-GGGCCATCCACAGTCTTCTG-3’
**SMAD1**	Forward	5’-TGCCACTCAACGCCACTTTT-3’
	Reverse	5’-TCATAAGCAACCGCCTGAACA-3’
**SMAD3**	Forward	5’-CGGCAGTAGATGACATGAGG-3’
	Reverse	5’-TCAACACCAAGTGCATCACC-3’
**TGFβ2**	Forward	5’-GGTACCTTGATGCCATCCCGCC-3’
	Reverse	5’-GCACTCTGGCTTTTGGGTTCTGCA-3’
**ALK1**	Forward	5’-TGGAGTGTGTGGGAAAAGGC-3’
	Reverse	5’-ATGTCAGTCTCCCGGAACCA-3’
**ALK5/TGFβR1**	Forward	5’-ACGGCGTTACAGTGTTTCTG-3’
	Reverse	5’-GCACATACAAACGGCCTATCT-3’
**CNN1**	Forward	5’-CTGGCTGCAGCTTATTGATG-3’
	Reverse	5’-CTGAGAGAGTGGATCGAGGG-3’
**SMA**	Forward	5’-CAAAGCCGGCCTTACAGA-3’
	Reverse	5’-AGGCCAGCCAAGCACTG-3’
**Col1A1**	Forward	5’-GAGGGCCAAGACGAAGACATC-3’
	Reverse	5’-CAGATCACGTCATCGCACAAC-3’
**ENG**	Forward	5’-CTGAGGACCAGAAGCACCTC-3’
	Reverse	5’-TCCATGTCCTCTTCCTGGAG-3’
**ID1**	Forward	5’-GCTGCTCTACGACATGAACG-3’
	Reverse	5’-CTCCAACTGAAGGTCCCTGA-3’

Supplementary Table 3. Human Oligonucleotides used in this study

Primers		Oligonucleotide Sequences
**ERG Ex6**	Forward	5’- CCGGATACTGTGGGGATGAG-3’
	Reverse	5’- TCTGCGCTCATTTGTGGTCA-3’
**HPRT**	Forward	5’- GTTAAGCAGTACAGCCCCAAAATG-3’
	Reverse	5’- TCAAGGGCATATCCAACAACAAAC-3’
**SMAD1**	Forward	5’-CCGAGCCGGCGCTAAC-3’
	Reverse	5’-GCTTCTTGGTCTGCGGTTTG-3’
**SMAD3**	Forward	5’-AAGAAGCTCAAGAAGACGGGG -3’
	Reverse	5’-CAGTGACCTGGGGATGGTAAT -3’
**TGFβ2**	Forward	5’-GGTACCTTGATGCCATCCCGCC-3’
	Reverse	5’-GCACTCTGGCTTTTGGGTTCTGCA-3’
**CNN1**	Forward	5’-TGGTGCCAGTTCTGAGTTGA-3’
	Reverse	5’-ATGTATGGCCTCAAAGACGG-3’

Supplementary Table 4. Mouse Oligonucleotides used in this study

Primers		Oligonucleotide Sequences
**CNN1 promoter region**	Forward	5’-CCGTGACTTCAGAGTCCGTG-3’
	Reverse	5’-CGTCACGCGATGACTCACT-3’
**CNN1 neg control region**	Forward	5’-ATGCTGGGAAGAAGGGGTGT-3’
	Reverse	5’-TACCTCCCCACCTGCCAAT-3’
**TGFβ2 promoter region**	Forward	5’-GCAGACACGTGGTTCAGAGA-3’
	Reverse	5’-AACATCACGATCTTGCCGGG-3’
**TGFβ2 neg control region**	Forward	5’-GTTATGAGTGGCCCAAAAGCC-3’
	Reverse	5’-CCACAGCATATCAGCAGGTTG-3’

Supplementary Table 5. Oligonucleotides used for ChIP-qPCR
